# Supplementary material for: Cryo-EM structure of a Ca2+-bound photosynthetic LH1-RC complex containing multiple αβ-polypeptides
Source: Nat Commun. 2020 Oct 2;11:4955. doi: 10.1038/s41467-020-18748-3 (PMC7532537; doi:10.1038/s41467-020-18748-3)
Supplement: Supplementary file 1 — Supplementary Information [file 41467_2020_18748_MOESM1_ESM.pdf]

SUPPLEMENTARY INFORMATION

Cryo-EM structure of a Ca^{2+}-bound photosynthetic LH1-RC complex containing multiple αβ-polypeptides

Kazutoshi Tani et al.

Supplementary Table 1 Cryo-EM data collection, refinement and validation statistics of the *Trv*. strain 970 LH1-RC complex

	LH1-RC complex (EMDB-30314) (PDB-7C9R)
**Data collection and processing**	
Magnification	96000
Voltage (kV)	300
Electron exposure (e–/Å^2)	40
Defocus range (μm)	-1.0 to -3.0
Pixel size (Å)	0.840
Symmetry imposed	C1
Initial particle images (no.)	153004
Final particle images (no.)	105234
Map resolution (Å)	2.8
FSC threshold	0.143
Map resolution range (Å)	336-2.8
**Refinement**	
Initial model used (PDB code)	5Y5S
Model resolution (Å)	2.9
FSC threshold	0.5
Model resolution range (Å)	139-2.8
Map sharpening *B* factor (Å^2)	-52
Model composition	
Non-hydrogen atoms	29228
Protein residues	3009
Ligands	124
B factors (Å^2)	
Protein	51.8
Ligand	65.1
R.m.s. deviations	
Bond lengths (Å)	0.016
Bond angles (°)	3.254
Validation	
MolProbity score	1.67
Clashscore	7.19
Poor rotamers (%)	0.43
Ramachandran plot	
Favored (%)	95.98
Allowed (%)	3.88
Disallowed (%)	0.14

Supplementary Table 2 Protein-protein interactions between RC and LH1-subunits in the transmembrane region within 3.5 Å.

RC-subunit	LH1-subunit
L-Asp21	α2-Arg19
L-Leu22	α2-Arg19
L-Trp26	α2-Arg20
L-Arg82	α2-Gly42
L-Gly19	α3-Ser20
L-Ala79	α3-Ser42
M-Leu109	α4-Gly42
M-Ser63	α4-Leu31
M-Leu53	α4-Arg20
M-Pro29	α4-Arg20
M-Gly80	α3-Ser42
M-Trp81	α3-Ser42
M-Asp82	α3-Ser42
M-Asp82	α3-Leu38
M-Glu69	α3-Phe31
M-Leu104	α1-Ser42

Supplementary Table 3 Comparison of the distances of His–BChl(Mg) and BChl(Mg)–BChl(Mg) in LH1, LH2 and RC special pairs from various phototrophic bacteria.

	Distance of His(Nε2) to BChl–Mg (Å)[a]		Distance of Mg–Mg (Å)[a]	
LH1 or LH2	α	β	Long	Short
Strain 970 (LH1)	**2.88**	**2.49**	**9.01**	**8.83**
Tch. tepidum (LH1)	2.19	2.19	8.88	8.72
Blc. viridis (LH1)	2.5	2.2	8.8	8.5
Rfx. castenholzii (B880)	2.3	2.3	9.5	9.3
Rps. acidophila (B850)	2.3	2.3	9.5	8.8
Phs. molischianum (B850)	2.3	2.3	9.2	8.9
Special pair in RC	L-subunit	M-subunit	BChl a_L–BChl a_M	
Strain 970	**2.55**	**2.58**	**7.92**	
Tch. tepidum	2.17	2.19	7.87	
Rba. sphaeroides	2.27	2.06	7.92	
Blc. viridis	2.36	2.35	7.83	

[a] These values were derived from Protein Data Bank: 5Y5S for *Tch. tepidum* LH1-RC, 6ET5 for *Blc. viridis* LH1-RC, 5YQ7 for *Rfx. castenholzii* B880, 1NKZ for *Rps. acidophila* LH2, 1LGH for *Phs. molischianum* LH2, 2J8C for *Rba. sphaeroides* RC.

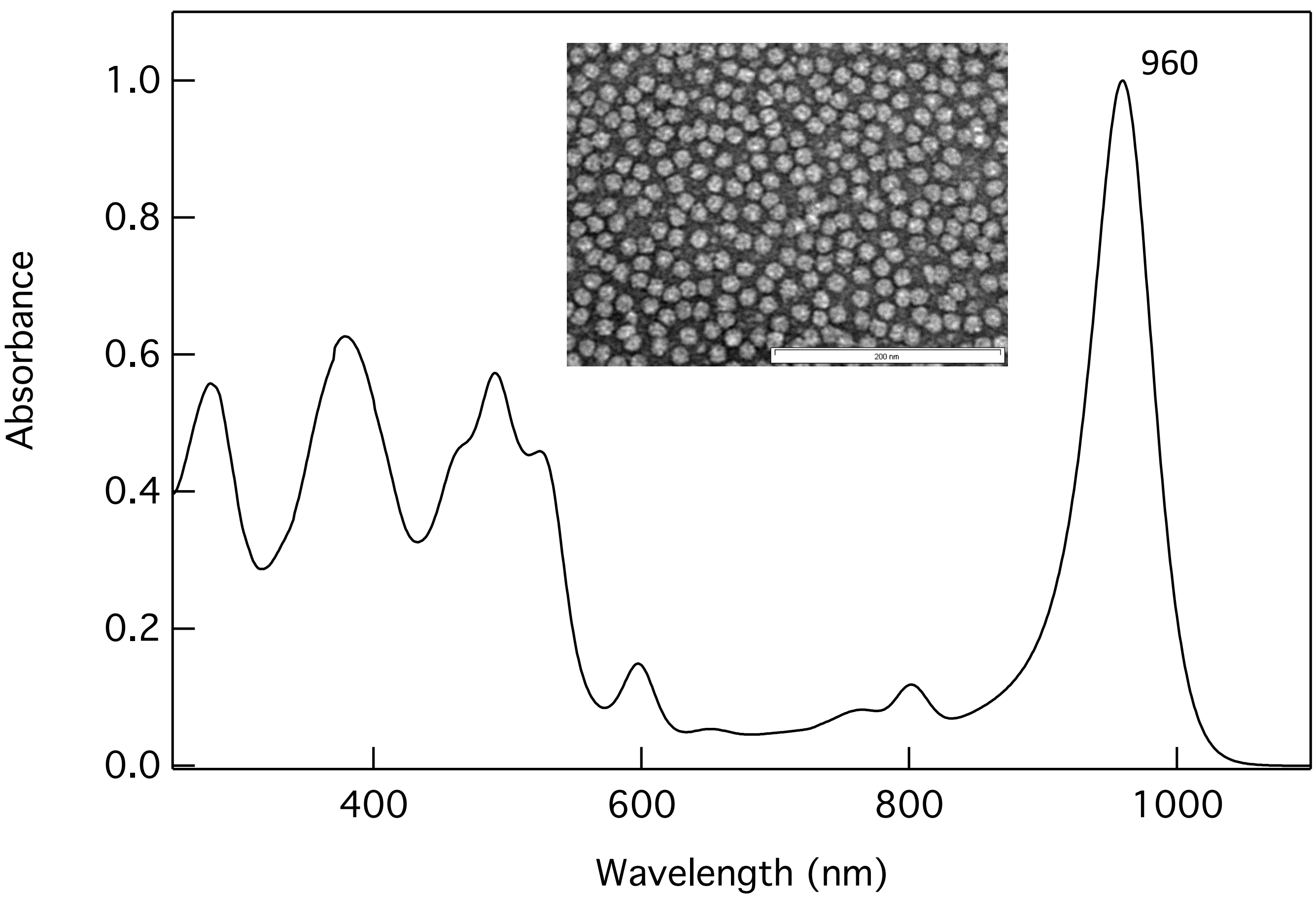

Supplementary Figure 1 Absorption spectrum of the purified *Trv.* strain 970 LH1-RC complex. Inset shows negatively stained LH1-RC particles obtained with 0.04 mg/mL LH1-RC in 20mM Tris-HCl (pH7.5), 0.05% DDM, 50 mM $CaCl_2$. Scale bar: 200 nm.

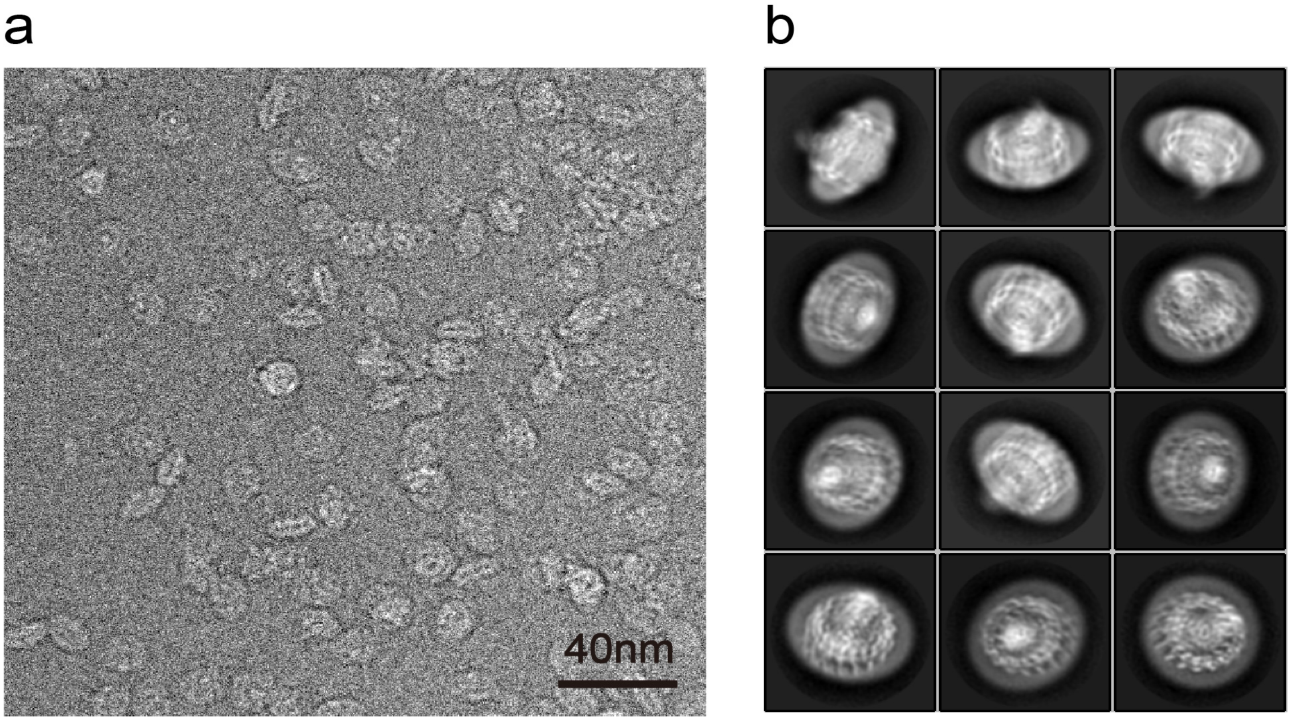

Supplementary Figure 2 Cryo-EM of the *Trv.* strain 970 LH1-RC complex. **(a)** A representative cryo-EM micrograph. **(b)** Representative 2D class averages processed from micrographs of LH1-RC.

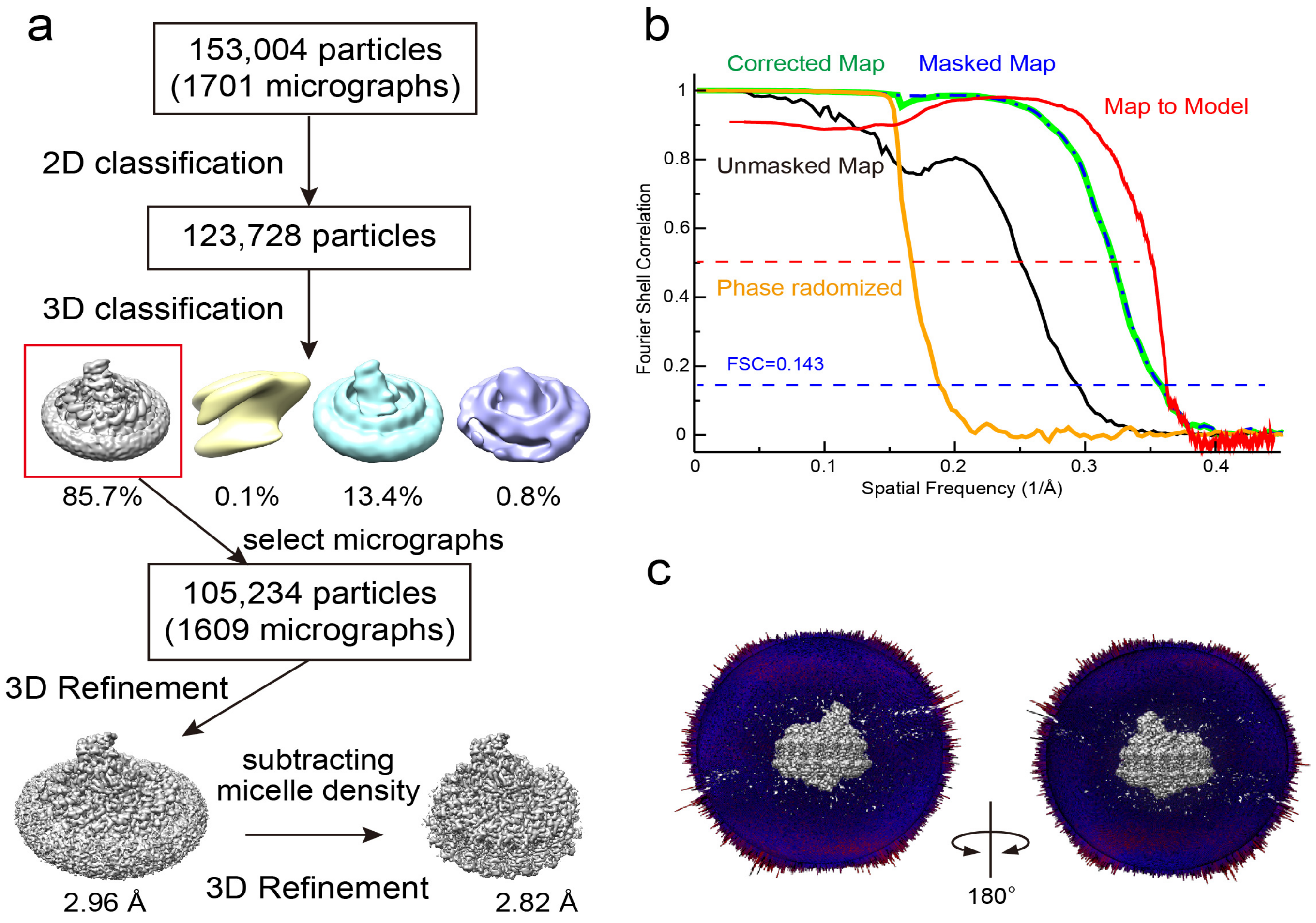

Supplementary Figure 3 Structure determination of the *Trv.* strain 970 LH1-RC complex. (a) Image processing flow of 3D classification and reconstruction. **(b)** The Fourier shell correlation (FSC) plots of the cryo-EM map (unmasked: black, masked: blue, phase randomized corrected: green, phase randomized: orange) and the FSC plot of the model versus the final map (red) are superimposed. **(c)** Angular distribution of reconstructed particles in the C1 map of LH1-RC complex. For clarity, the front half of angular distribution is removed.

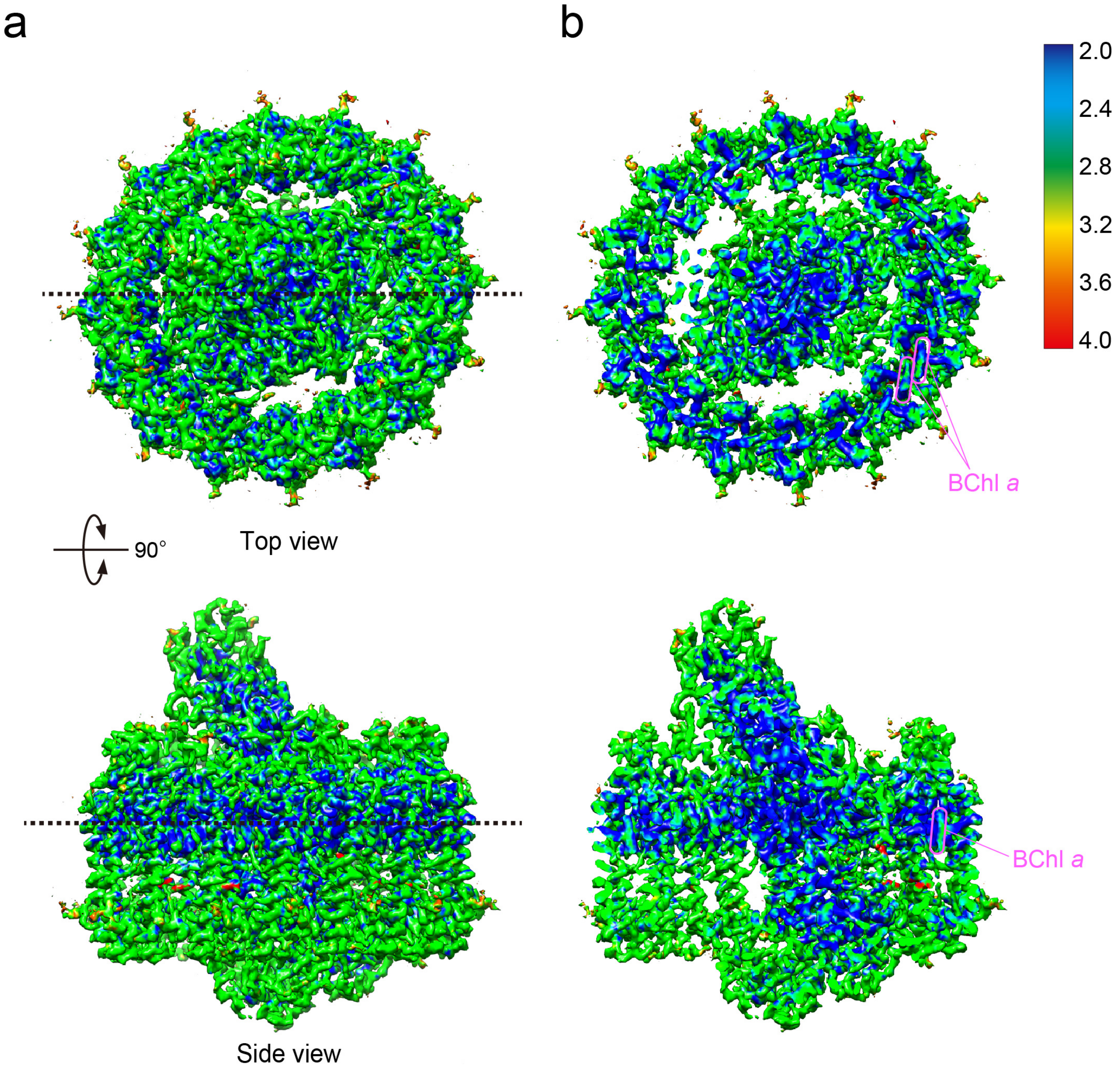

Supplementary Figure 4 Local resolution representation of the structure of LH1-RC complex. **(a)** Top view from periplasmic side and side view parallel to the membrane plane. Each dotted line indicates the cross section line. **(b)** A central cross sectional view of the left side of the panel. A region indicated by circular pink line corresponds to BChl *a*. The map is shown in the colors of the rainbow according to the estimated resolution from 4.0 Å (red) to 2.0 Å (blue).

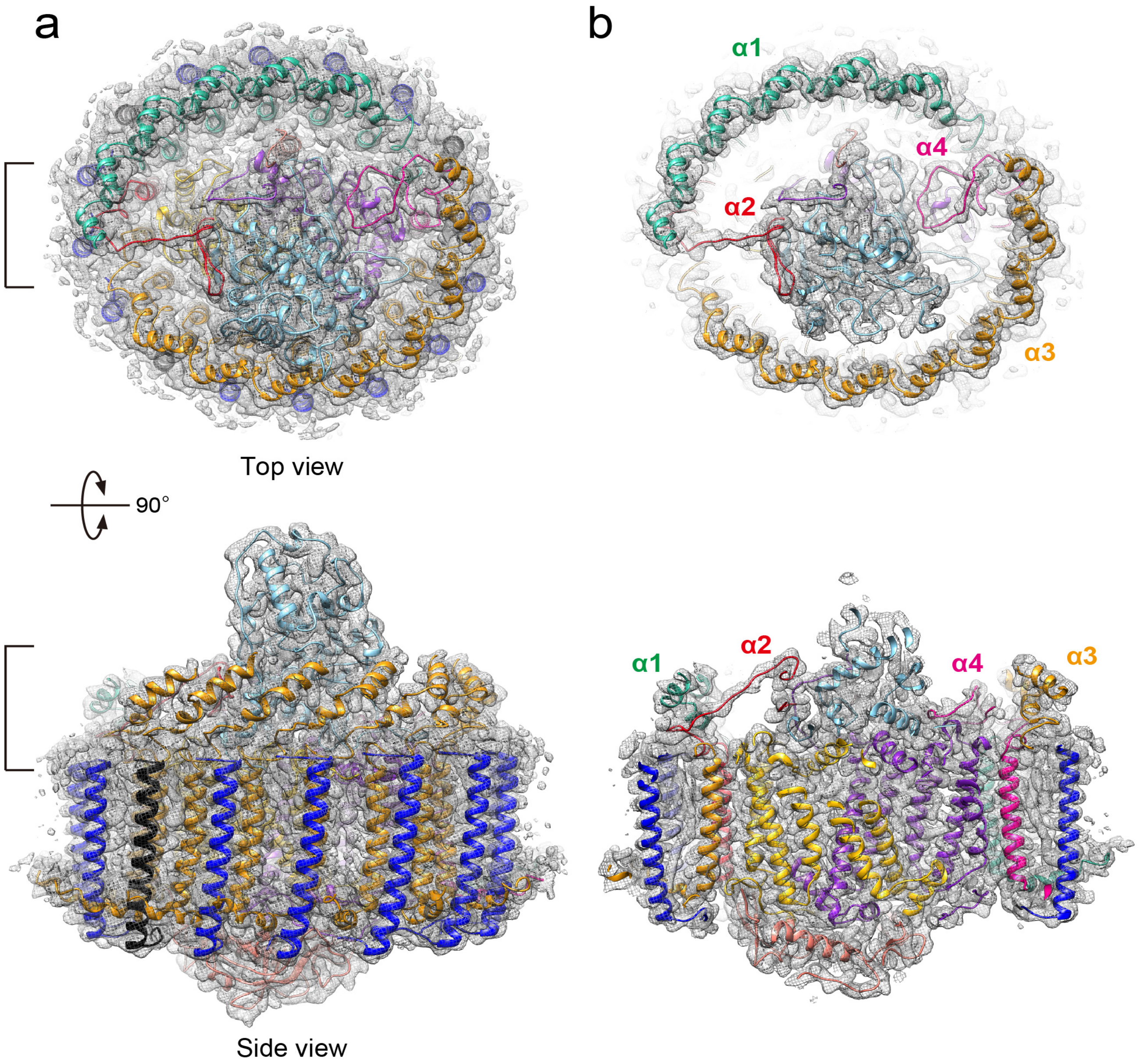

Supplementary Figure 5 Overall structure of the *Trv.* strain 970 LH1-RC complex. Cartoon representation of the complex with the cryo-EM density shown in grey mesh. **(a)** Top view from periplasmic side and side view parallel to the membrane plane. Each bracket indicates the slab region. **(b)** Top and side views of the slab along and perpendicular to the membrane plane. The color codes corresponding to polypeptides and pigments are the same as in Fig. 3 except the RC. The RC L-, M-, C-, H-subunits shown by light orange, blue violet, cyan, salmon pink, respectively.

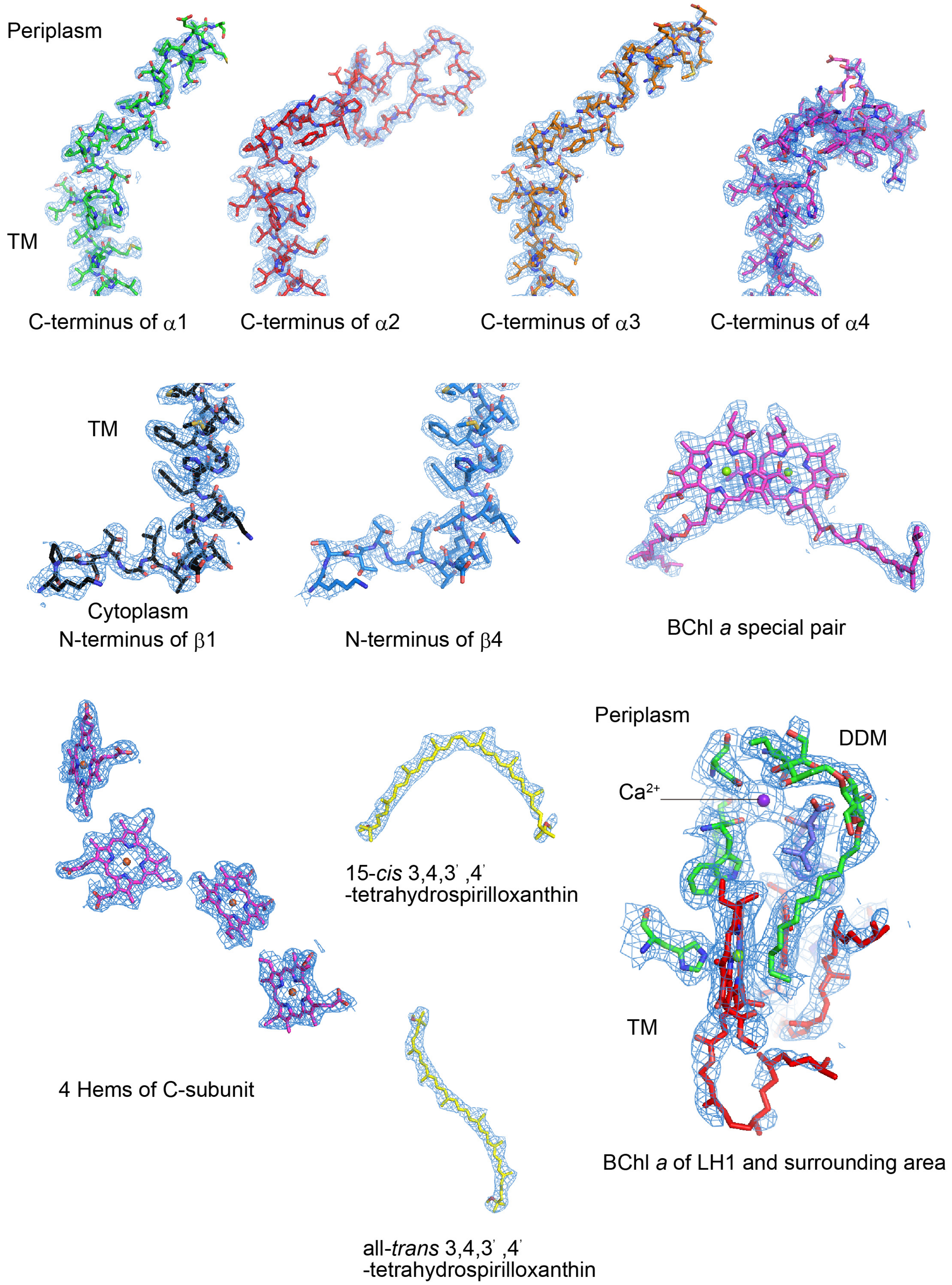

Supplementary Figure 6 Cryo-EM densities and structural models in the *Trv.* strain 970 LH1-RC complex. The color codes of polypeptides and pigments are the same as in Figs. 3 and 1, respectively. The density map is shown at a contour level of 4.0σ.

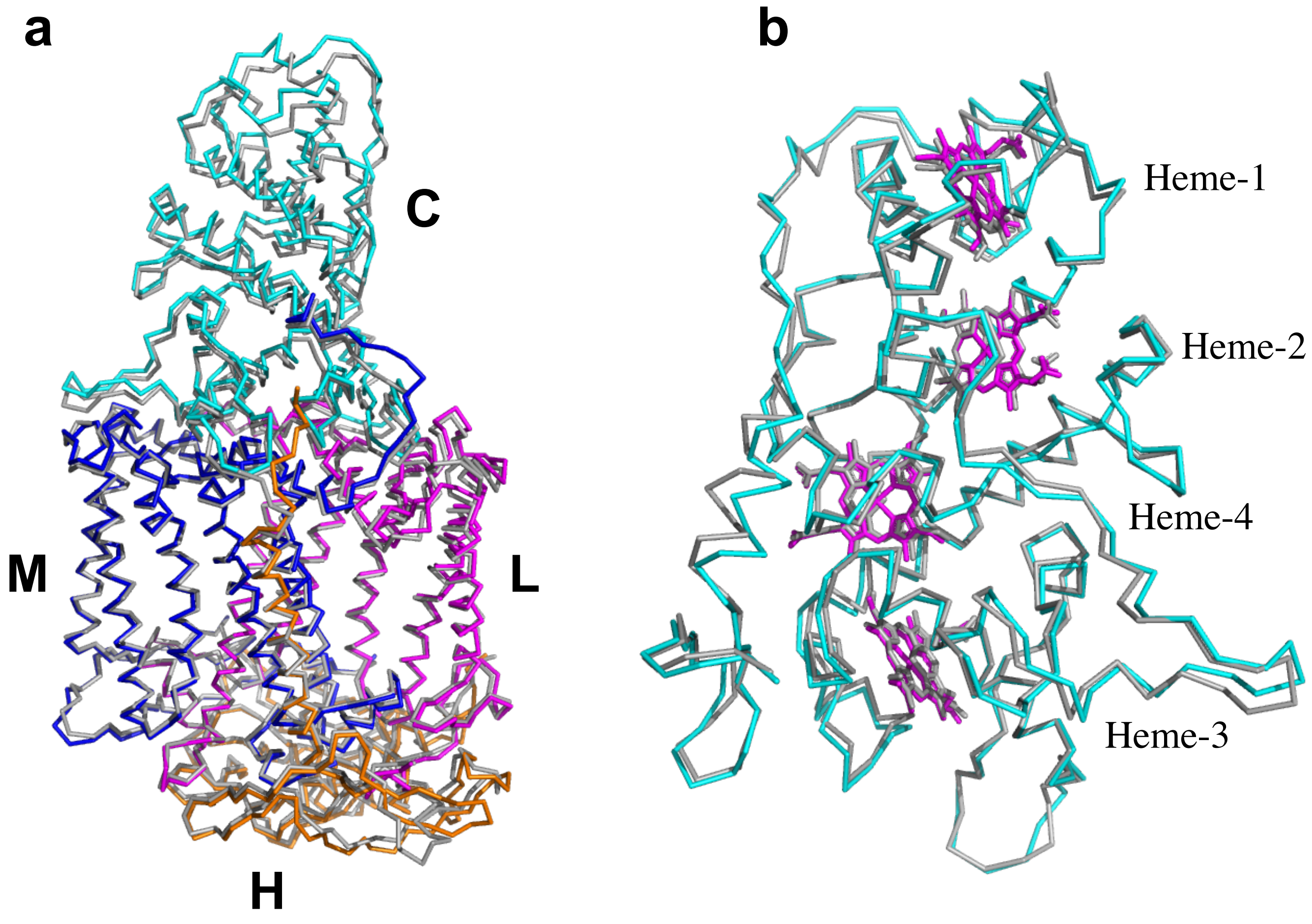

Supplementary Figure 7 Comparison of arrangement of the RC subunits between *Trv.* strain 970 and *Tch. tepidum* (grey, PDB ID: 5Y5S). **(a)** Side view of alignment of the RC polypeptides by superimposing the Cα carbons of the RC M-subunit. The *Trv.* strain 970 RC chains are colored in cyan for C-subunit, magenta for L-subunit, blue for M-subunit and orange for H-subunit. The root-mean-square deviations (RMSD) for the Cα carbons are 0.800 Å for C-subunits, 0.651 Å for L-subunits, 0.670 Å for M-subunits and 0.682 Å for H-subunits when aligned for each subunit pair. **(b)** Alignment of the Cα carbons of the RC C-subunits with heme groups. The heme groups of *Trv.* strain 970 are shown in magenta sticks.

a

```
                          10          20          30         40  ↓         50          60          70
Strain 970 α1   MNAKSFDGMHKLWMIMNPVSTLWAIFIFQIFLGLLIHMVVLSS-DLNWHDDQIPVGYQLQGETLPVNLEMKAAQ
Tch. tepidum    -MFTMNANLYKIWLILDPRRVLVSIVAFQIVLGLLIHMIVLST-DLNWLDDNIPVSYQALGKK-----------
Alc. vinosum    ----MSPDLWKIWLLVDPRRILIAVFAFLTVLGLAIHMILLSTAEFNWLEDGVPAA------------------
                          .: *:*::::*   * ::. *  .*** ***::**: ::** :* :*..
```

b

```
            10          20          30          40          50          60          70          80
α1 MNAKSFDGMHKLWMIMNPVSTLWAIFIFQIFLGLLIHMVVLSSDLNWHDDQIPVGYQLQGETLPVNLEMKAAQ
α2 MNSDKFAGMYKLWTFIDPRRTLIFIVAFQIMLGILIHMIVLGSDLNWHNDGIPRFYSPRPVDVAVGPAGIPLEIPGSPMPQARNYN
α3 MNAKSFDGMHKLWMIMNPVSTLWAIFIFQIFLGLLIHMVVLSSDLNWHDDQIPVGYQLQGETLPVNLEMKAALKDAQ
α4 MSNDNFTGTYKMWMFIDPRRALLFIASFQILLGILIHMIVLGSDLNWHSDGIPKFYFPNAAEASAPIDMSPIPSARNFKFD
α5 MHKVWKMIDPGRVVLTIFGFQAMLGLLVHSIVLGADPNWPDDGSAPISSPSEMLSELDPKNQSERLAR

β1 MAEKPSTGLTESEAKEFHGLFMASMTLWFGLVVLAHILSWMYRPWL
β2 MAEKTATGLTESEAKEFHGLFMASMTLWFGLVVLAHILSWLYRPWL
β3 MAEKSTTGLTEAESKEFHELFMASMTLWFGLVVLAHVLSWMYRPWL
β4 MAEKSTTGLTEAESKEFHGIFMASMTLWFGLVVLAHILSWLYRPWL
```

c

CLUSTAL 2.1 Sequence alignment score:

	α2	α3	α4
α1	47	99	42
α2		44	59
α3			40

d

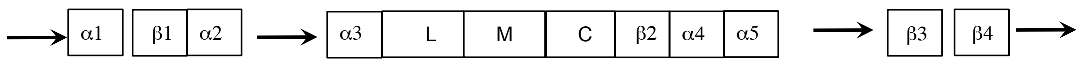

Supplementary Figure 8 Sequence information of LH1 polypeptides. (a) Sequence comparison of the LH1 α-polypeptides between purple sulfur bacteria *Trv*. strain 970 (α1), *Tch*. *tepidum* and *Alc*. *vinosum* (α1). The sequences are aligned relative to the BChl *a*-coordinating histidine residues (magenta). Residues in red fonts form hydrogen bonds with BChl *a* C3-acetyl group. Vertical arrow indicates deletions in *Trv*. strain 970 and *Tch*. *tepidum*. The BChl *a*-coordinating His is shown in magenta, and the hydrogen-bonding Trp and His are shown in red. Symbol scheme: (*) identical, (.) similar, (:) highly similar. **(b)** Amino acid sequences of the *Trv*. strain 970 LH1 α- and β-polypeptides from the genome database (GenBank assembly accession: GCA_000228725.3). The polypeptides and some N-terminal Met residues shown in grey color were not identified in the purified protein. The residues in the red boxes were used for assignments of the multiple polypeptides. **(c)** Comparison of the sequence similarity between the LH1 α-polypeptides (α1~ α4) using the CLUSTAL 2.1. **(d)** Arrangement of the gene cluster coding for the LH1 polypeptides and RC L-, M- and C-subunits in the genome.

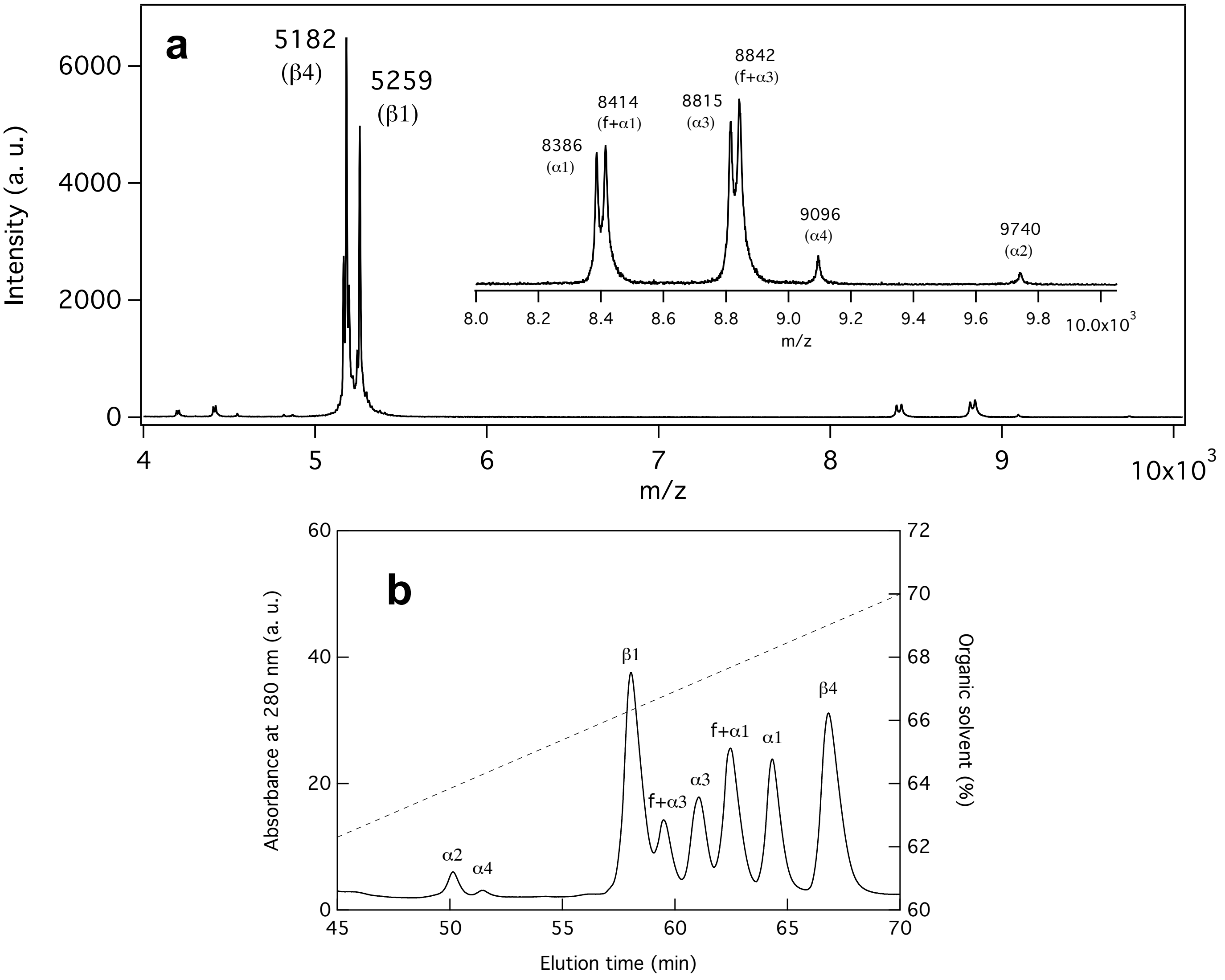

Supplementary Figure 9 Identifications of the multiple LH1 polypeptides. The letter "f" denotes formylation. **(a)** MALDI-TOF /MS spectrum of the purified LH1-RC obtained under the same conditions as described in Ref. 42. Inset: expanded region of the m/z = 8000–10000. **(b)** Reverse-phase HPLC chromatogram (TSKgel, SuperODS, 4.6×100 mm, TOSOH) of the purified LH1-RC eluted at 25 ˚C by a gradient of 50–70% organic solvent consisted of acetonitrile/2-propanol (2:1) containing 0.1% trifluoroacetic acid.

L-subunit

```
                150       160       170       180       190       200
S970_L          GLFSHLDWVSNVGYQTLHFHYNPAHMLAISFFFINTLALAMHGSLILSVVNPQKGEEVKT
TTP_L           GILSHLDWVSNVGYQFLHFHYNPAHMLAISFFFTNCLALSMHGSLILSVTNPQKGEPVKT
Rbs_L           GIWTHLDWVSNTGYTYGNFHYNPAHMIAISFFFTNALALALHGALVLSAANPEKGKEMRT
B_viridis_L     GILSHLDWVNNFGYQYLNWHYNPGHMSSVSFLFVNAMALGLHGGLILSVANPGDGDKVKT
                *: :*****.* **   ::****.** ::**:* * :**.:**.*:**..** .*. ::*

                210       220       230       240       250       260
S970_L          AEHENTVFRDIVGYSIGALAIHRLGLFLAINAAFWSAVCMILTGPFWTRGWPEWWMWWPN
TTP_L           SEHENTFFRDIVGYSIGALAIHRLGLFLALSAAFWSAVCILISGPFWTRGWPEWWNWWLE
Rbs_L           PDHEDTFFRDLVGYSIGTLGIHRLGLLLSLSAVFFSALCMIITGTIWFDQWVDWWQWWVK
B_viridis_L     AEHENQYFRDVVGYSIGALSIHRLGLFLASNIFLTGAFGTIASGPFWTRGWPEWWGWWLD
                .:**:  ***:******:*.******:*: .  : .*.  : :*.:*   * :** ** .
```

M-subunit

```
                180       190       200       210       220       230
S970_M          FPHLDWTAAISIRYGNFYYNPFHGLSIAFMYGSAVLFAMHGGTILAVSRYGGDREIDQIT
TTP_M           FPHLDWTAAFSIRYGNLYYNPFHMLSIAFLYGSALLFAMHGATILSVSRFGGDREIDQIT
Rbs_M           FSHLDWTNNFSLVHGNLFYNPFHGLSIAFLYGSALLFAMHGATILAVSRFGGERELEQIA
B_viridis_M     WPHIDWLTAFSIRYGNFYYCPWHGFSIGFAYGCGLLFAAHGATILAVARFGGDREIEQIT
                :.*:**   :*: :**::* *:* :**.* **..:*** **.***:*:*:**:**::**:

                240       250       260       270       280       290
S970_M          DRGTAAERAMLFWRWCMGFNASMESIHRWAWWFAVFCIINSILGIILTGTVVDNWYLWAV
TTP_M           HRGTAAERAALFWRWTMGFNVTMESIHRWAWWCAVLTVITAGIGILLSGTVVDNWYLWAV
Rbs_M           DRGTAAERAALFWRWTMGFNATMEGIHRWAIWMAVLVTLTGGIGILLSGTVVDNWYVWGQ
B_viridis_M     DRGTAVERAALFWRWTIGFNATIESVHRWGWFFSLMVMVSASVGILLTGTFVDNWYLWCV
                .****.*** ***** :***.::*.:***. : :::  :.. :**:*:**.*****:*
```

Supplementary Figure 10 Comparisons of partial amino acid sequences of the RC L- and M-subunits between *Trv.* strain 970 (S970), *Tch. tepidum* (TTP), *Rba.*) *sphaeroides* (Rbs) and *Blc. viridis* (B_viridis). The special pair BChl *a*-coordinating His residues are shown by the red bold fonts. The residues interacting with the special pair BChls *a* are shown in magenta and marked in the red boxes. Symbol scheme: (*) identical, (.) similar, (:) highly similar.

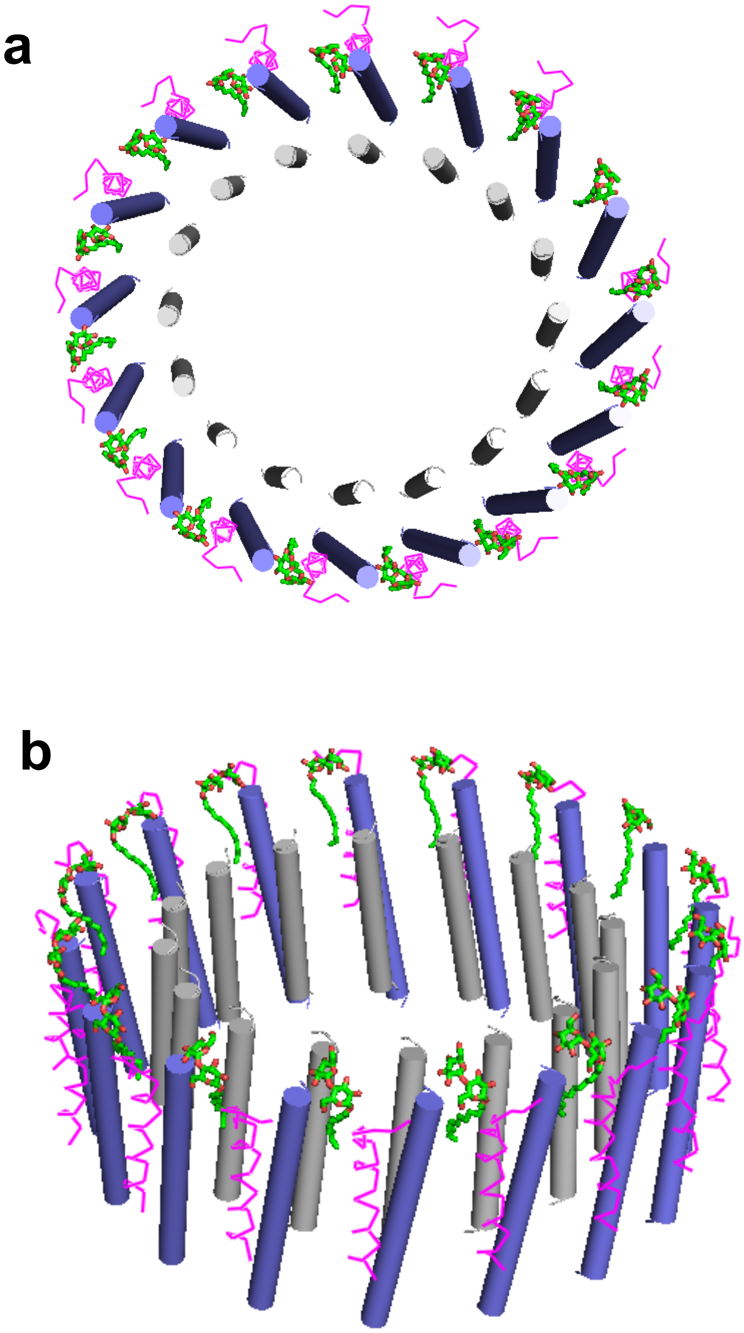

Supplementary Figure 11 Illustration of the relative positions of the DDM molecules (green sticks) in the *Trv.* strain 970 LH1 and those of the LH1 γ-polypeptides (magenta ribbons) in *Blc. viridis* (PDB ID: 6ET5). The two structures were superimposed with their LH1 α β-polypeptides, and the LH1 α- and β-polypeptides of *Blc. viridis* were omitted for clarity. **(a)** Top view from periplasmic side. **(b)** Tilted view.

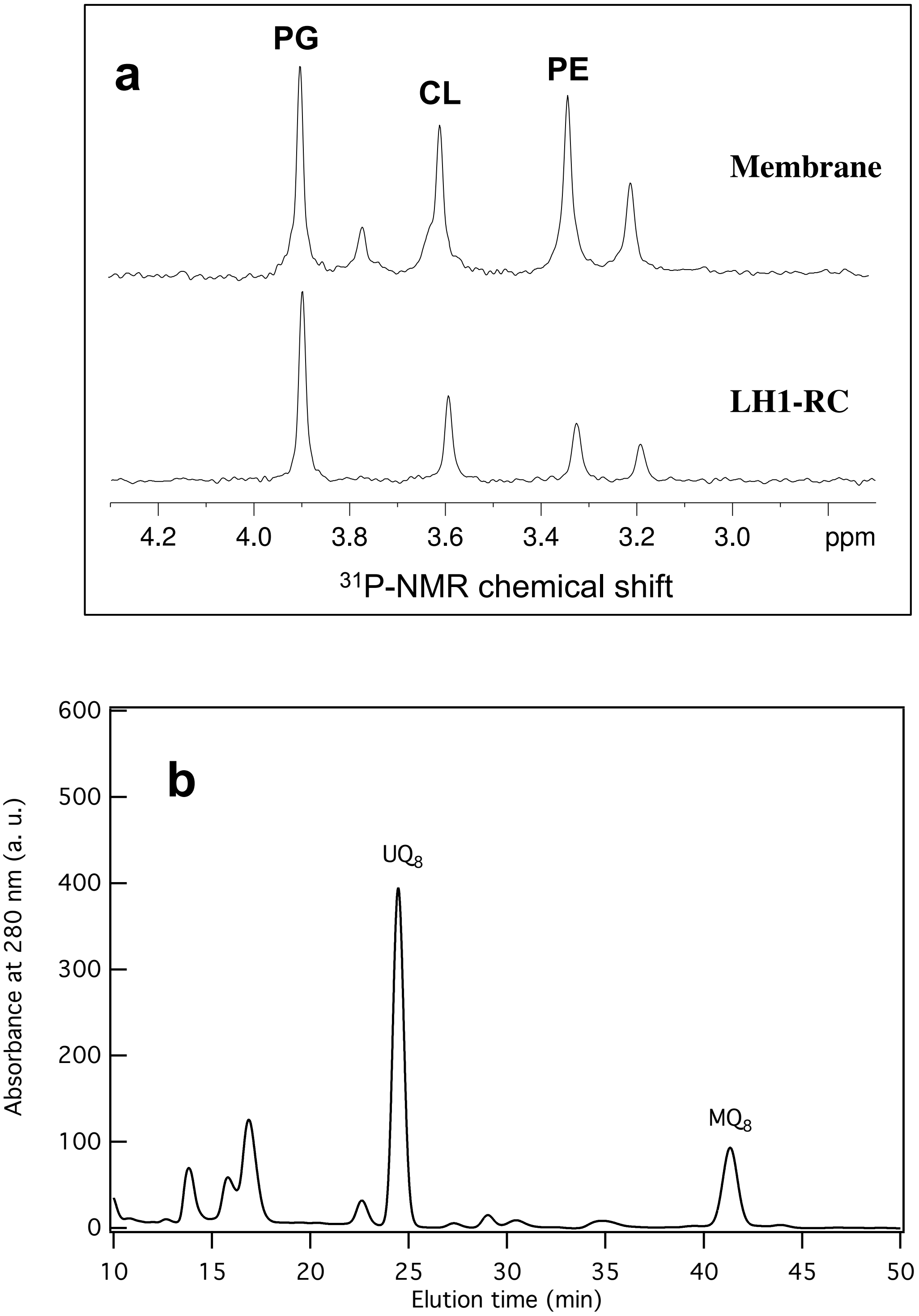

Supplementary Figure 12 Identifications of the phospholipids and quinones in the purified *Trv.* strain 970 LH1-RC. (a) ^{31}P-NMR spectra of the membrane and purified LH1-RC obtained under the same conditions as described in Ref. 27. **(b)** Reverse-phase HPLC chromatogram (TOSOH, TSKgel ODS-80Ts, 4.6×250 mm) of the extracted quinones and pigments isocratically eluted at 25 ˚C by 7:3 methanol/isopropanol at flow rate of 0.7 mL/min.

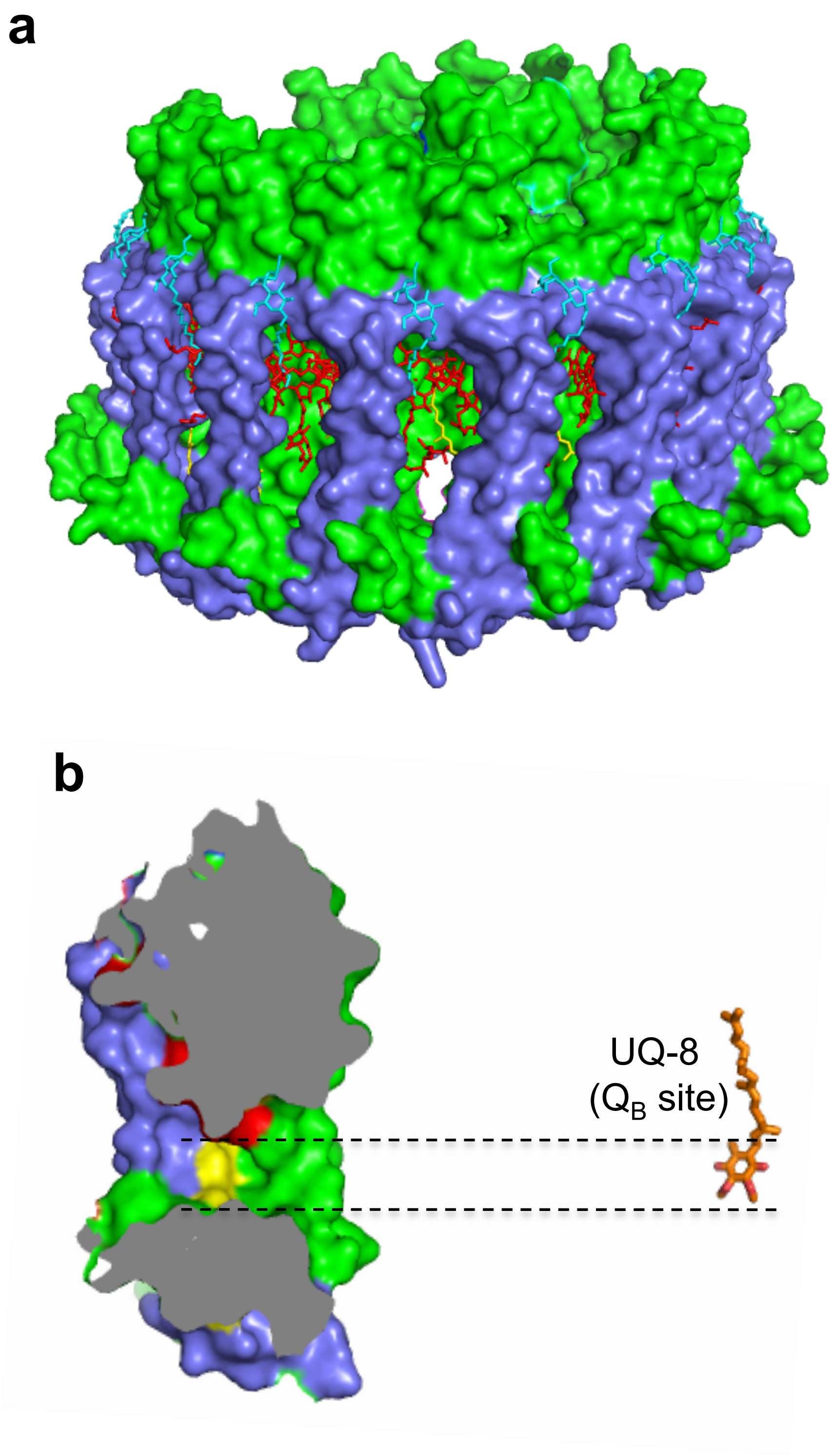

Supplementary Figure 13 Channels in the LH1 ring. (a) Surface representation of the *Trv.* strain 970 LH1 complex. Major cofactors are shown by sticks: BChl *a* (red), carotenoid (yellow) and DDM (cyan). **(b)** Cross section of a putative quinone channel illustrating the channel opening and the size of the benzoquinone head group of the ubiquinone-8 (orange sticks) at Q_B site. Cofactors are also shown in surface representation.
